# Involvement of energy metabolism in the production of ‘bystander effects’ by radiation

**DOI:** 10.1054/bjoc.2000.1109

**Published:** 2000-04-27

**Authors:** C Mothersill, T D Stamato, M L Perez, R Cummins, R Mooney, C B Seymour

**Affiliations:** Radiation Science Centre, Dublin Institute of Technology, Kevin Street, Dublin 8, Ireland; Lankenau Medical Research Center, 100 Lancaster Avenue, Wynnewood, PA, 19096, USA

**Keywords:** radiation-induced bystander effect, mitochondria, epigenetic radiation mechanisms

## Abstract

These experiments were done to determine if interference with energy metabolism and REDOX biochemistry during low LET radiation exposure would alter the ability of medium harvested from the irradiated cells to induce a bystander effect in unirradiated cells. Human keratinocyte cells and CHO-K1 mutant cell lines were irradiated using cobalt 60. Clonogenic assays were used to determine the reproductive death of the cells exposed to direct irradiation or medium from irradiated cells. The persistence in progeny was also examined. Use of apoptosis inhibitors or medium from the LDH or G6PD null cell lines, reduced or prevented the bystander effect. Transfection with G6PD recovered the effect. Treatment with anti-oxidant substances, L -lactate and L -deprenyl prevented bystander factor associated cell kill. The lactate analogue, oxamate, was less effective. Data from experiments where media harvested from the different cell lines was exchanged suggest that signal production and cellular response may involve different mechanisms. The effects on exposed cells were transmitted to progeny which also showed excessive levels of cell death for several generations. The results suggest that energy/REDOX metabolism may be involved in the expression of a radiation induced bystander response. Given the aberrant energy metabolism in tumour cells, this may have implications for dose escalation in radiotherapy. © 2000 Cancer Research Campaign


*British Journal of Cancer* (2000) **82**(10), 1740–1746
© 2000 Cancer Research Campaign
DOI: 10.1054/ bjoc.2000.1109, available online at http://www.idealibrary.com on IDEAL®

### Involvement of energy metabolism in the production of 'bystander effects' by radiation

**C Mothersill[1], TD Stamato[2], ML Perez[2], R Cummins[1], R Mooney[1] and CB Seymour[1]**

[1]Radiation Science Centre, Dublin Institute of Technology, Kevin Street, Dublin 8, Ireland; [2]Lankenau Medical Research Center, 100 Lancaster Avenue, Wynnewood, PA 19096, USA

**Summary** These experiments were done to determine if interference with energy metabolism and REDOX biochemistry during low LET radiation exposure would alter the ability of medium harvested from the irradiated cells to induce a bystander effect in unirradiated cells. Human keratinocyte cells and CHO-K1 mutant cell lines were irradiated using cobalt 60. Clonogenic assays were used to determine the reproductive death of the cells exposed to direct irradiation or medium from irradiated cells. The persistence in progeny was also examined. Use of apoptosis inhibitors or medium from the LDH or G6PD null cell lines, reduced or prevented the bystander effect. Transfection with G6PD recovered the effect. Treatment with anti-oxidant substances, L-lactate and L-deprenyl prevented bystander factor associated cell kill. The lactate analogue, oxamate, was less effective. Data from experiments where media harvested from the different cell lines was exchanged suggest that signal production and cellular response may involve different mechanisms. The effects on exposed cells were transmitted to progeny which also showed excessive levels of cell death for several generations. The results suggest that energy/REDOX metabolism may be involved in the expression of a radiation induced bystander response. Given the aberrant energy metabolism in tumour cells, this may have implications for dose escalation in radiotherapy. © 2000 Cancer Research Campaign

**Keywords:** radiation-induced bystander effect; mitochondria; epigenetic radiation mechanisms

There is considerable evidence to suggest that mitochondria are central in initiating the apoptotic cascade (Kroemer et al, 1996, 1997; Zamzami et al, 1996; Kluck et al, 1997; Green et al, 1998). In this paper we examine the effects of aberrant energy metabolism and modulation of apoptosis on 'bystander' cell killing following irradiation. 'Bystander' damage is the term used to describe the killing or damaging of cells not directly hit by radiation. Several papers have appeared recently which suggest that a factor or signal can be produced by cells irradiated with high or low LET radiation, which can affect cells which are in the field but not hit or which are not even in the field (Nagasawa and Little, 1994; Mothersill and Seymour, 1996, 1997*a*, 1998*a*; Deshpande et al, 1997; Lorimore et al, 1998). These effects are not limited to the first few generations of un-hit cells but the descendants of these cells can produce progeny showing classic evidence of genomic instability (Seymour and Mothersill, 1997; Lorimore et al, 1998). The effects often appear to saturate at relatively low doses (0.05–1 Gy gamma or alpha irradiation) and occur very quickly, even within 15 min of exposure (Mothersill and Seymour, 1997*a*). The worldwide results to date do suggest that different cell types express different levels or characteristics of this effect and it is too early to define general characteristics (Deshpande et al, 1997; Assam and Little, 1998; Lorimore et al, 1998; Mothersill and Seymour, 1998). The low LET effect has mainly been examined using cell death as an end point and apoptosis is a predominant mechanism in both normal and tumour cells which show a bystander effect. Data from alpha irradiated cells shows higher than expected expression of chromosomal aberrations (Lorimore et al, 1998) and sister chromatid exchanges (SCEs), (Nagasawa and Little, 1994) in the un-hit cells. Lorimore et al (1998) show convincing evidence that the genomic instability is associated with the un-hit rather than the hit cells in their culture system. Work by Emerit and colleagues (Emerit et al, 1967) found evidence for a clastogenic factor in the plasma of irradiated individuals which could produce excess levels of SCEs and they suggest a role for oxidative stress in the mechanism. This is supported by recent data from Clutton et al (1996) showing that irradiated survivors which carry genomic instability have higher than normal oxygen radical production.

Oxidative stress-related effects often involve oxidative energy metabolism and experiments by Mothersill and Seymour (1998*a*) showing suppression of production of the factor by low temperature suggest that the bystander effect is energy-dependent. For this reason it was decided to see if interference with the central energy metabolic pathway of glycolysis, or with the mitochondrially located hexose monophosphate (HMP) shunt would alter the extent of the bystander effect.

Glycolytic energy metabolism can be affected in many ways but the most suitable methods for these experiments were to load human keratinocyte cells with lactate or its non-active analogue oxamate. Previous work by this laboratory had already established the toxicity and radiobiological effects of these compounds (Seymour and Mothersill, 1981, 1985, 1987, 1988; Mothersill and Seymour, 1986). Lactate alters oxidative metabolism by loading the cell with reduced NAD and this can cause product feedback inhibition of the steps leading to pyruvate production. Oxamate is not metabolized by LDH and competes with lactate naturally occurring in the cell, so a metabolic effect of lactate would be reduced by oxamate. If purely chemical radical scavenging were the basis of a lactate-mediated alteration in bystander cell death

*Received 5 October 1999*
*Revised 10 January 2000*
*Accepted 19 January 2000*

*Correspondence to:* C Mothersill then both agents should have the same effect. This point was also examined using mutant CHO-K1 cells with no lactate dehydrogenase. Direct involvement of the HMP shunt was examined, using CHO-K1 mutants with no glucose-6-phosphate dehydrogenase (G6PD) or mutant (non-functional) G6PD. The possibility of an apoptosis associated mechanism was tested directly using an ICE inhibitor (Ac-YVAD-Cmk) that inhibits the activity of selected caspases directly involved in the apoptotic cascade and cyclosporin A which prevents the change in mitochondrial membrane potential, thought to be a key factor in the initiation of apoptosis (Hockenberry et al, 1990; Jacobsen et al, 1993; Kumar 1995; Nicholson et al, 1997; Villa et al, 1997; Thornberry and Lazebnik, 1998). The effect of L-deprenyl, which appears to prevent apoptosis by up-regulating bcl-2 (Mothersill et al, in preparation) was also examined.

### METHODS

### Cell lines

A human keratinocyte cell line supplied as a gift by J Di Paolo (NIH, Bethesda, MD, USA) was used for experiments unless otherwise indicated. This line was originally immortalized by transfection with the HPV 16 (Pirisi et al, 1988). It is p53 null due to expression of E6 protein by the virus but grows in culture to form a characteristic monolayer of 'cobblestone-like' keratinocytes. These display contact inhibition and gap junction communication. For experiments with the glycolytic and HMP shunt pathways a series of lines donated by co-author Stamato was used. These were developed from the CHO-K1 line. The mutants used in this paper are LDH null, which does not express LDH, E48 which expresses mutant G6PD, E89 which is G6PD null and E89t, which is the null cell line with the G6PD gene transfected back in. Full details of the cell lines can be found in Stamato et al (1987).

#### *Cell culture*

All cell lines were maintained in an incubator at 37°C in an atmosphere of 5% carbon dioxide ($CO_2$) in air. HPV keratinocytes were cultured in Dulbecco's modified Eagles medium (DMEM):F12 (1:1) containing 7% fetal calf serum (FCS), 5 ml penicillin streptomycin solution, 25 mM HEPES buffer and 1 μg ml$^{-1}$ hydrocortisone (all from Gibco Biocult Ltd, Irvine, UK). CHO-K1 derived cell lines were grown in Nutrient Mixture, F12 (Ham) containing 10% FCS, penicillin–streptomycin solution and 25 mM HEPES buffer, except where media exchange experiments were planned. In this case CHO lines were adapted to the keratinocyte medium for 2 months before use. All lines were used for experiments when sub-confluent. Subculture was routinely performed using a 1:1 solution of 0.25% trypsin and 1 mM EDTA in Earle's balanced salt solution at 37°C. HPV keratinocytes required incubation for approximately 10 min before detachment occurred, CHO lines detached in 2–4 min.

#### *Clonogenic assays*

Subconfluent flasks that had received a medium change the previous day were chosen. Cells were removed from the flask using trypsin–EDTA solution. When the cells had detached they were resuspended in medium, syringed gently to produce a single cell suspension and an aliquot was counted using a Coulter counter model $D_N$ set at a threshold calibrated for each cell line using a haemocytometer. Appropriate cell numbers were plated for survival using the clonogenic assay technique of Puck and Marcus (1956). Flasks destined to donate medium were plated with cell numbers in the region of $2 \times 10^5$. Medium was harvested 1 h post-irradiation, which took place 6 h after plating. The harvested medium was transferred to cultures containing cloning densities of cells (approx. 120 cells for CHO lines and approx. 600 for HPV-G cells) set up at the same time as the donors.

Controls for irradiation of medium only were included in each experiment. Controls for transfer of unirradiated medium from densely seeded cultures to cultures seeded at cloning densities were also always included as were simple plating efficiency controls. Cultures were incubated in 5 ml of culture medium in 25 cm$^2$, 40 ml flasks (Nunclon, Denmark), in a humidified 37°C incubator in an atmosphere of 5% $CO_2$ in air. After 7 days (CHO lines) or 12 days (HPV-G line), cultures were stained with carbol fuchsin and macroscopic colonies were counted. The percentage survival was corrected for the appropriate control plating efficiency. For assay of delayed expression of cell death in progeny cells, the technique established in Seymour et al (1986) was used. This involves growing cultures to confluence (approx 15 further cell doublings) instead of staining them for colony counting. A clonogenic assay is then performed on the culture at this stage. A reduction in clonogenic survival in the progeny of clonogenic survivors is considered to be an end point of genomic instability in cells and is evidence of induction of delayed damage (Mothersill and Seymour, 1998*b*).

#### *Medium transfer*

The technique used has been described in detail in Mothersill and Seymour (1997). Briefly, medium was poured off donor flasks 1 h after irradiation. The medium was filtered through a 0.22 μ filter used to sterilize solutions, to ensure that no cells could still be present in the transferred medium. This was confirmed by examination of aliquots of medium under the microscope. Culture medium was then removed from the flasks designated to receive irradiated medium and the filtrate was immediately added to these recipient flasks. A medium change of unirradiated but similarly filtered medium from unirradiated donor flasks seeded at the donor density of approx. 300 000 cells per flask was given to controls at the same time. Standard plating efficiency controls were also set up. There was never a significant difference between these two controls. Standard clonogenic survival points following irradiation were also always included, with and without a medium change at the appropriate time. No effect of changing the medium was found for any of the cell lines. The donor medium generated as described in this paragraph is referred to as ICM (irradiated conditioned medium).

#### *Irradiation*

Cultures were sealed and irradiated at room temperature using a cobalt 60 teletherapy unit delivering approximately 2.0 Gy min$^{-1}$ during the time period of these experiments. The source to flask distance was 80 cm and the field size was $30 \times 30$ cm. Flasks were returned to the incubator immediately after irradiation.

#### *Preparation of chemicals*

All chemicals were prepared as concentrated (at least 50 ×) aqueous solutions. Where it was necessary to dissolve a compound, dimethyl sulphoxide (DMSO) was used and a dilution of at least 1:1000 was done before the addition of the aliquot to the flask. DMSO at these concentrations was included in the control.

© 2000 Cancer Research Campaign

**Table 1A** Effect of irradiation in the presence of lactate (10 mM) or oxamate (10 mM) on the cloning efficiency of directly irradiated or ICM-treated cells. Corrected PE values are shown with the actual PE values for controls in brackets

| Treatment | Control | Lactate | Oxamate |
|---|---|---|---|
| 0 Gy | 100 (20.9 ± 0.9) | 100 (21.9 ± 0.2) | 100 (22.5 ± 0.87) |
| ICM from 0 Gy | 100 (22.2 ± 1.0) | 100 (19.4 ± 2.4) | 100 (20.2 ± 0.78) |
| 5.0 Gy | 20.4 ± 1.2 | 15.9 ± 3.2 | 27.7 ± 0.9 |
| ICM from 5 Gy | 64.9 ± 3.6 | 98.2 ± 8.1 | 83.3 ± 6.7 |

**Table 1B** Effect of irradiation in the presence of lactate (10 mM) or oxamate (10 mM) on the cloning efficiency of progeny of directly irradiated or ICM-treated cells. PE values are corrected to the appropriate control

| Treatment | Control progeny | Lactate progeny | Oxamate progeny |
|---|---|---|---|
| 0 Gy | 100 (27.1 ± 3.7) | 100 (22.2 ± 1.2) | 100 (30.19 ± 1.8) |
| ICM from 0 Gy | 100 (27.7 ± 1.9) | 100 (25.9 ± 1.7) | 100 (29.6 ± 7.5) |
| 5.0 Gy | 62.7 ± 10.5 | 112 ± 3.5 | 79.2 ± 6.0 |
| ICM from 5 Gy | 68.9 ± 4.2 | 109 ± 8.6 | 71.0 ± 6.9 |

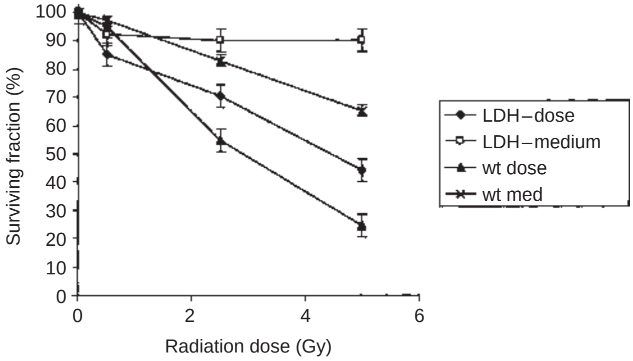

**Figure 1** Reduction in the clonogenic survival of CHO-KI parent cells (WT) and those which are LDH null following direct irradiation or exposure to ICM. Errors are the s.e.m. for *n* = 9

Solutions were filtered using 0.22-μ filters and then diluted in culture medium so that the required concentration could be added to the medium in the flask as a 1:50 aliquot. Substances used were L-lactate (hemicalcium salt, Sigma, London, UK), sodium oxamate (Sigma Aldrich, Poole, UK), ICE inhibitor (Ac-YVAD-Cmk) (Calbiochem-Novabiochem, UK), cyclosporin A (Sigma Aldrich, Poole, UK), and L-deprenyl (obtained as a gift from the Department of Biochemistry, Trinity College, Dublin, Ireland). For bystander experiments, the relevant chemical was added (except where indicated), to the recipient flasks, control flasks and the donor flasks at the same time to standardize exposure.

### Statistical analysis

All experiments were repeated three times. Three replicates were counted for each experimental point in each experiment to assess the surviving fraction. The data are presented as mean ± standard error in all cases. Where significance was assessed, a *t*-test was used.

### RESULTS

### Modification of LDH activity

Table 1a shows the effect of direct irradiation or treatment with ICM on the survival of HPV-G cells exposed to 10 mM L-lactate or L-oxamate. The effect on the progeny of these cells is shown in Table 1b. It can be seen that treatment of cells with L-lactate prevents the reduction in plating efficiency (bystander effect) in populations of cells receiving ICM. Oxamate does not prevent the bystander effect but reduces the level of cell killing seen in the controls. These effects persisted in the distant (at least 15 cell population doublings) progeny of progenitors which survived exposure to irradiated medium. In Table 1 it is apparent that the delayed effects under each treatment condition are not significantly different in progeny of directly irradiated or ICM-treated cells. Since loading a cell with lactate will drive LDH in the direction of pyruvate with consequent reduction of NAD, the data suggested a role for lactate dehydrogenase, or for oxidized REDOX substrates in the mechanism, therefore an LDH null line of CHO-K1 cells was tested. In this line there was only a very small reduction (not statistically significant) in clonogenic survival of ICM-treated cultures (Figure 1), while a reduction in survival of ICM-treated CHO-K1 parent cell line of up to 40% was found ($P < 0.001$). The possibility that REDOX balance was involved, together with the observation that apoptosis is a prominent mechanism of cell killing following ICM treatment (Mothersill and Seymour, 1997), suggested that oxidative biochemistry might be important in the initiation of the bystander effect. This was explored further in the next section.

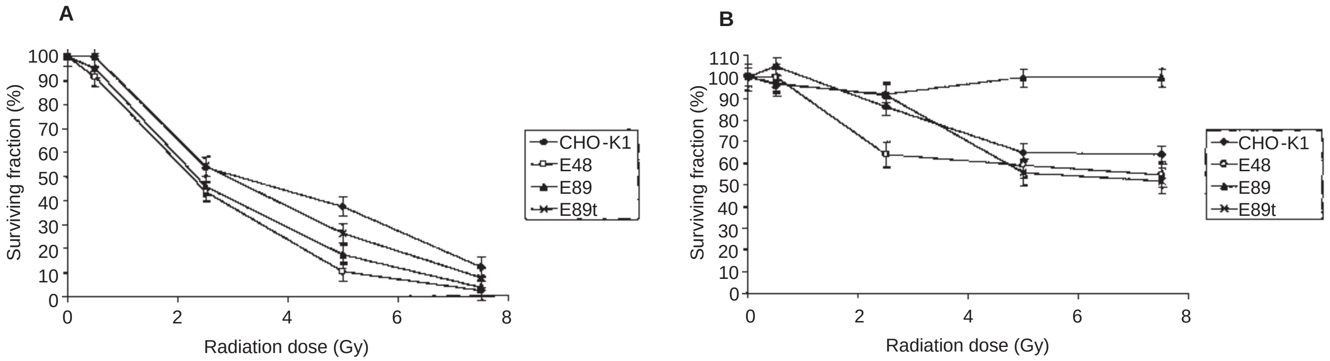

**Figure 2** The reduction in clonogenic survival following direct irradiation (**A**) or exposure to medium from irradiated cells with specific mutations in the G6PD enzyme (**B**). Errors are the s.e.m. for *n* = 9

*© 2000 Cancer Research Campaign*

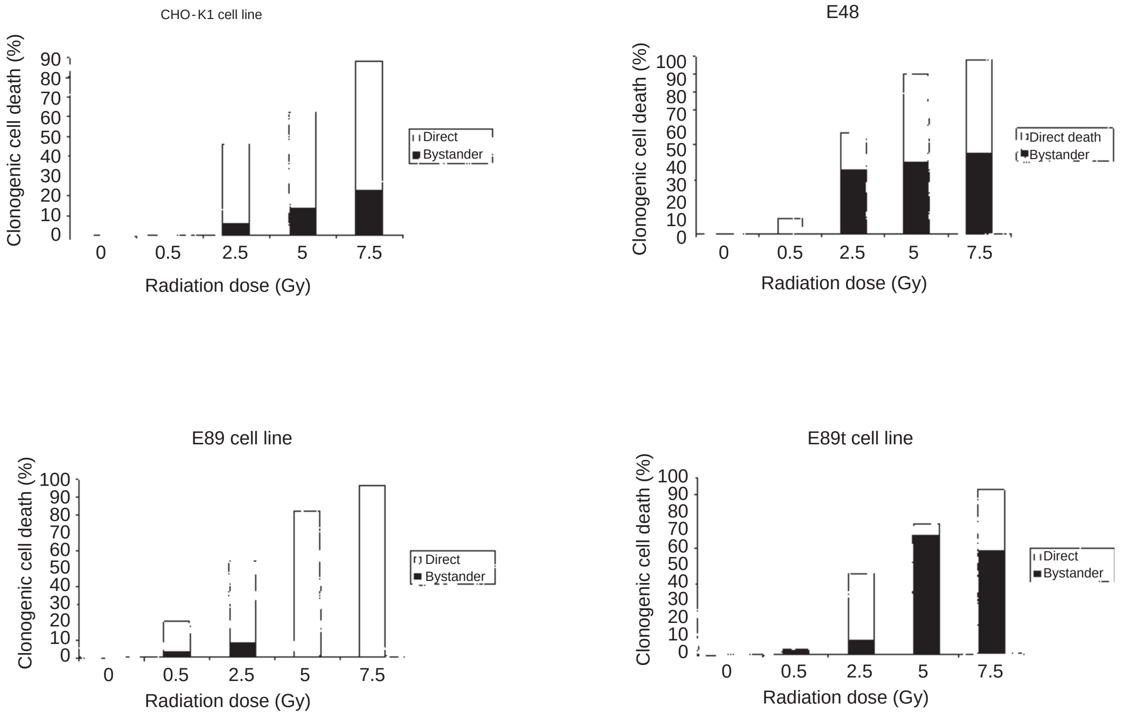

**Figure 3** Clonogenic cell death plots for CHO-K1, E48, E89 and E89t cell lines showing the relative contribution of direct radiation interaction with cells and the killing due to bystander effects in cells not directly hit. Mean data for three separate experiments with three replicate flasks in each experiment

### Use of cell lines with abnormal oxidative metabolism

Figure 2 shows the extent of reduction in clonogenic survival following exposure to a direct dose or to medium from irradiated cells with specific mutations in the G6PD enzyme. The effect of direct irradiation is shown in Figure 2A. The lines show slightly different radiosensitivities with the CHO-K1 parent line being the most radioresistent but the differences in radiosensitivities are not statistically significant. The effect of treatment with ICM is shown in Figure 2B for each of these lines. The parent CHO-K1 line demonstrates a bystander effect ($P < 0.01$ at 5 Gy). The E48 line which expresses G6PD but produces a protein which is abnormal also demonstrates bystander cell killing. For these lines the level of cell survival post-ICM exposure appears to plateau at about 60% ($P < 0.01$). The E89 line which is G6PD null has no bystander activity but transfection of the G6PD gene into the null line regains the bystander effect at the level found in the parent line. The results show that almost all the cell death occurring in irradiated E89 cells is due to direct irradiation, but in the other lines up to 40% of the clonogenic death is attributable to bystander effects. The data are presented as histograms in Figure 3. These are plotted by subtracting the % of cells killed by exposure to ICM from the total cells killed by exposure to the dose. This gives the amount of cell death attributable to direct interaction of radiation with cells if bystander induced killing in the flask is corrected out.

Table 2 shows the delayed effects of direct irradiation of progenitor cells on the progeny of these cell lines tested by performing a clonogenic assay on cells grown from colonies of survivors which have undergone about 15 cell population doublings. Clearly in the CHO-K1, E89t and E48 lines there is a long-term effect, which is transmissible to progeny and very similar in magnitude to the initial effect induced by ICM exposure. There is, however,

**Table 2** Plating efficiencies (corrected to 100% for each control) of the progeny of G6PD mutant cells which had undergone at least 15 cell population doublings post irradiation. The progenitor cells were exposed to direct irradiation

| Dose (Gy) | CHO-K1 | E48 | E89 | E89T |
|---|---|---|---|---|
| 0 | 100 (74.3 ± 4) | 100 (57.6 ± 4) | 100 (46.1 ± 2) | 100 (47.2 ± 6) |
| 0.5 | 101 ± 3.3 | 66.5 ± 3.7 | 87.9 ± 3.7 | 93.9 ± 6.1 |
| 2.5 | 73.7 ± 6.6 | 64.3 ± 4.9 | 82.6 ± 3.1 | 81.1 ± 7.6 |
| 5.0 | 72.7 ± 5.4 | 53.4 ± 3.1 | 86.8 ± 2.5 | 85.0 ± 5.9 |
| 7.5 | 64.1 ± 3.8 | 49.2 ± 3.3 | 62.3 ± 3.8 | 71.0 ± 6.3 |

**Table 3** The effects of putative inhibitors of apoptosis initiation in mitochondria on PE ± s.e.m. of directly irradiated HPV G cells and recipients of ICM. Corrected PE values are shown with the actual PE values for controls in brackets

| Treatment | Direct dose | ICM |
|---|---|---|
| Control 0 Gy | 100 (16.8 ± 0.8) | 100 (17.3 ± 1.5) |
| Control + 5 Gy | 21.5 ± 3.2 | 56.1 ± 9.4 |
| 20 μM ICE inhibitor (ICEh) | 100 (16.2 ± 2.5) | 100 (16.7 ± 1.4) |
| 5 Gy (20 μM ICEh to donor cells) | 42.3 ± 7.1 | 72.9 ± 12.3 |
| 5 Gy (20 μM ICEh to recipient cells) | N/A | 89.2 ± 7.9 |
| 0.3 μM cyclosporin A (cyA) | 100 (13.9 ± 1.0) | 100 (14.7 ± 1.5) |
| 5 Gy (0.3 μM cyA to donor cells) | 34.8 ± 4.0 | 49.5 ± 7.8 |
| 5 Gy (0.3 μM cyA to recipient cells) | N/A | 75.2 ± 8.3 |
| 9 nM L-deprenyl (Ldep) | 100 (27.1 ± 3.4) | 100 (25.6 ± 2.5) |
| 5 Gy + 9 nM Ldep to donor cells | 38.2 ± 4.7 | 66.7 ± 2.1 |
| 5 Gy + 9 nM Ldep to recipient cells | 43.2 ± 2.6 | 81.2 ± 2.9 |

*© 2000 Cancer Research Campaign*

**Table 4** Effect of exchange of media between cell lines exhibiting differing responses to irradiated conditioned medium. Each % PE was corrected for control PE using a similarly treated flask of cells which was not irradiated Mean ± s.e.m. for $n = 3$

| Medium donor cell line | Medium recipient cell line | % PE of recipient |
|---|---|---|
| E89 | E89 | 103 ± 4.8 |
| E89t | E98t | 49.9 ± 6.2 |
| E89 | HPV-G | 54.0 ± 3.7 |
| E89t | HPV G | 58.5 ± 7.1 |
| E89 | E89t | 61.3 ± 6.1 |
| E89t | E89 | 98.8 ± 5.9 |

evidence of delayed death in the E89 line and the result for this line is in the same range as is found for the parent line.

### Effect of inhibitors of apoptosis

A major mechanism of cell death due to ICM is apoptosis (Mothersill and Seymour, 1997). In Table 3 the effect of substances which prevent or delay apoptosis through pathways involving oxidative metabolism are shown. Cyclosporin A inhibits the collapse of the mitochondrial membrane potential (Kroemer et al, 1997) and this prevents initiation of apoptosis. The ICE inhibitor (AC-YVAD-Cmk) also delays or reduces apoptosis. It is predominantly effective on caspase I but also inhibits caspase III to a lesser extent. L-deprenyl is an anti-oxidant that has an obscure effect on the mitochondrial part of the apoptotic pathway that involves up-regulation of Bcl-2 expression. It can be seen that these substances also reduce or prevent the bystander effect. The effect of cyclosporin A or L-deprenyl added to the donor cells is more effective in reducing survival than if the substances are added to the ICM after harvest but in the case of ICE inhibitor, the addition is equally effective whether the donor cells or only the recipients are treated.

### Separation of the signal generation from the cellular response

As a consequence of the data presented here and earlier experiments by the group involving exchange of medium between cell lines (Mothersill and Seymour, 1997), it was decided to examine the possibility that the bystander factor might be produced in cell lines which did not respond to it. In Table 4, results are presented where media is exchanged between the mutant E89 and E89t lines and also where ICM from these lines is transferred to a different responding line (keratinocytes). The data show that if media from irradiated E89 cells (which have no bystander effect), and from irradiated E89t cells (which have regained a bystander effect) are exchanged, the E89 cells are able to produce the factor but do not respond to it. Where media from both cell lines are transferred to unirradiated keratinocyte cells which normally show a bystander effect, both mutant lines produce the signal/factor which leads to clonogenic cell death in the keratinocyte line. This suggests that the signal generation is distinct and under independent control from the response to the signal.

### DISCUSSION

The effect of lactate and oxamate on the production of a bystander effect was tested because the effect of these substances on the response of cells to direct irradiation was like the bystander effect, i.e. predominantly a low dose effect which changed the shape of the survival curve shoulder. Seymour and Mothersill in a series of papers showed that adding lactate, oxamate, glycolysis inhibitors or analogues of glucose to CHO cells had complex effects on their radiation response (Seymour and Mothersill, 1981; Seymour, 1983; Seymour et al, 1985; Mothersill and Seymour, 1986; Seymour and Mothersill, 1987, 1988). They concluded that the key factor determining the response of the cells was the ability to produce ATP as an energy source for repair, repopulation or programmed cell death. Lactate used shortly before irradiation was radioprotective while oxamate was not. The results in this paper are consistent with the earlier findings and confirm that addition of lactate to the medium within 6 h of irradiation is radioprotective. This radioprotection may be due to abolition of the bystander effect. Oxamate is an analogue of lactate which cannot be metabolized by LDH and by competing with lactate, it delays regeneration of $NAD^+$ from the LDH-mediated reaction under anaerobic conditions. This compound reduces the bystander effect and the clonogenic survival is intermediate between the control and the lactate-treated points. These data suggest that generation of ATP or generation of critical REDOX potential may be critical to the production of a bystander effect. ATP would be required for any energy dependent process and the NAD/NADH balance is critical for the continued progress of metabolism.

As an approach to determining whether energy metabolism per se is critical, cells which were LDH null were examined. The LDH null cells were found to have no bystander effect. Metabolism of glucose to lactate (anaerobic glycolysis) is a major form of ATP production by CHO cells cultured in an atmosphere of 5% $CO_2$ in air. Seymour (1983) clearly demonstrates this by measuring glucose use and lactate production during growth of these cells by showing almost stoiciometric equivalence of lactate to glucose in culture medium during growth of cells. Thus the lack of a bystander effect in LDH null cells is further evidence that the breakdown of glucose to produce lactate is associated with the production of a bystander effect. Another major pathway which produces energy and reduced substrates to drive metabolism is the oxidative HMP shunt (discussed in Schwartz, 1997). The data presented in this paper show that the bystander effect is not present in the CHO-derived cell line (E89) which is null for G6PD. This enzyme is rate-limiting for the mitochondrially located HMP shunt. The bystander effect is regained when the DNA sequence coding for the G6PD enzyme is reintroduced. It is interesting that the E48 mutant CHO line which produces defective G6PD protein has a bystander effect equivalent to that of the parent line. This suggests that the active site for the HMP shunt role of G6PD which

© 2000 Cancer Research Campaign converts G6P to 6-phosphoglucono-δ-lactone with the generation of NADPH+ $H^+$ may be different to that needed to transduce the bystander effect.

These data all suggest that generation of ATP or reduced NAD/NADP may be critical to the production of a bystander effect. ATP would be required for any energy dependent process but since the bystander effect is detected by our system as a response which involves an increase in apoptosis (Mothersill and Seymour, 1998), we decided to test the effect of inhibitors of the initiation of apoptosis. It was found that preventing apoptosis using caspase inhibitor, cyclosporin A or deprenyl all stopped the bystander effect. A problem exists with experiments where inhibitors which may have many cellular effects are claimed to provide evidence of involvement of a particular pathway. This is the reason why three agents that affect apoptosis in completely different ways were used. The ability of all to modulate the effect is strong evidence for the involvement of energy-dependent initiation of apoptosis in the bystander pathway.

The data in this paper raise questions for radiobiology, particularly concerning mechanisms of low-dose radiation-induced cell death and the importance of direct DNA strand-break damage in this process. A problem arises with the data in Figures 2 and 3. The effect of direct irradiation as usually calculated gives very similar survival curves for each cell line but the bystander effect is clearly different for the E89 cells. The plot in Figure 3 is generated by subtracting the bystander component of cell death from the total death to give the amount of death which can be attributed to direct irradiation alone. This plot shows that while the total amount of death induced by a dose is relatively constant for each line and is clearly a function of dose, the mechanism of death (direct or bystander), is different and is not a function of dose in that the bystander component tends to saturate. This means that the 'target' concept of a deposition of energy causing physical damage in the DNA (see Hall, 1994), may not be the predominant mechanism of death at low doses and further suggests that bystander effects may predominate at these doses. Consideration of delayed death provides further evidence for these suggestions. The amount of delayed cell death is very similar to the amount of bystander death. An exception to this is the obvious induction of delayed death in E89 cells which have no bystander effect. An explanation for this is not immediately obvious but it must be remembered that death is only one end point of the bystander effect, and delayed death is only one end point of genomic instability. Lorimore et al (1998), showed evidence of instability induced in bystander cells using non-clonal chromosome aberrations as an end point, Wu et al (1999), show evidence, using a microbeam, of higher than expected levels of mutations in cell populations where the cytoplasm but not the nucleus was hit. It is possible that E89 cells generate a signal that does not result in initial cell death, perhaps because the cells cannot undergo the cell death process induced by the factor, but that it still induces genomic instability resulting in delayed death and other effects. The relationship between genomic instability endpoints is still far from clear.

A key point in this discussion is that the generation of a bystander signal is most likely a different process from the expression of a death response. Early work by our group on the bystander effect showed that while a fibroblast cell line (MSU 1) showed no bystander effect, medium from an irradiated epithelial line killed the fibroblast cells (Mothersill and Seymour, 1997). Other work by Lorimore et al (1998) showed that the cells which demonstrate instability following alpha-particle irradiation are not those which received the hit. These data clearly suggest that the signal generation is separate from the response. In this paper, this distinction is also apparent; because medium from irradiated E89 cells and E89t cells both produced a bystander effect when ICM was transferred to unirrradiated HPV cells even though the unirradiated E89 cells which received medium from irradiated E89 cells showed no effect. Clearly, the ability of E89 cells to die in response to the signal is compromised by the lack of G6PD but the signal production is present. This distinction may be important in efforts to determine the mechanisms involved in bystander effects and of course has major implications for radiotherapy, where tumours often have poor apoptotic responses relative to surrounding normal tissue. Given the results for the apoptosis inhibiting substances, it is likely that the integrity of the apoptotic response is essential for the expression of the killing response in affected cells but perhaps not for the generation of the signal in the irradiated cells. This will also have important implications for mechanistic studies involving multi cell type tissues or models. It may also help in the elucidation of the pathways involved in genomic instability (Morgan et al, 1996). Morgan points out that the current dogma that radiation-induced mutations, however delayed, are somehow due to the initial mutation is hard to reconcile with the facts of the instability phenotype (high frequency, non-clonal aberrations and high levels of excess division failure in distant progeny). Many of the unusual characteristics of the genomic instability dose–response curve may be more easily understood if it is assumed that the phenotype results from a signalling mechanism induced in one cell by irradiation which affects others in its vicinity. Cytokine or stress responses mediated by biochemical signalling substances and receptors are common tissue responses to DNA damaging agents or oxy-radicals. Whatever the mechanisms, the existence of a bystander effect may have implications for our current understanding of low-dose effects in radiobiology. It may also be relevant to the determination of factors controlling normal tissue response to radiotherapy.

### ACKNOWLEDGEMENTS

We acknowledge, with sincere thanks, the support of the Irish Cancer Research Advancement Board who funded this work. We also thank Saint Luke's Hospital for access to their Cobalt 60 therapy source.

### REFERENCES

Clutton SM, Townsend KMS, Walker C, Ansell JD and Wright EG (1996) Radiation induced genomic instability and persisting oxidative stress in primary bone marrow cultures. *Carcinogenesis* **17**: 1633–1639

Deshpande A, Goodwin EH, Bailey SM, Marrone BL and Lehnert BE (1997) Alpha-particle-induced sister chromatid exchange in normal human lung fibroblasts: evidence for an extra-nuclear target. *Radiat Res* **145**: 260–267

Emerit I, Filipe P, Meunier P, Auclair C, Freitas J, Deroussent A, Gouyette A and Fernandes A (1997) Clastogenic activity in the plasma of scleroderma patients: a biomarker of oxidative stress. *Dermatology* **194**: 140–146

Green DR and Reed JC (1998) Mitochondria and apoptosis. *Science* **281**: 1309–1312

Hall EJ (1994) *Radiobiology for the Radiologist*, 4th edn. Lippencott and Lippencott: Philadelphia

Hockenberry D, Nuñez G, Milliman C, Schreiber RD and Korsmeyer SJ (1990) Bcl-2 is an inner mitochondrial membrane protein that blocks programmed cell death. *Nature* **348**: 334–336

Jacobson MD, Burne JF, King MP, Miyashita TM, Reed JC and Raff MC (1993) Bcl-2 blocks apoptosis in cells lacking mitochondrial DNA. *Nature* **361**: 365–369

*© 2000 Cancer Research Campaign* 

Kluck RM, Bossy-Wetzel E, Green DR and Newmeyer DD (1997) The release of cytochrome c from mitochondria: a primary site for Bcl-2 regulation of apoptosis. *Science* **275**: 1132–1136
Kroemer G, Zamzami N and Susin SA (1997) Mitochondrial control of apoptosis. *Immunol Today* **18**: 44–51
Kumar S (1995) ICE-like proteases in apoptosis. *Trends Biochem Sci* **29**, 198–202
Lehnert BE and Goodwin EH (1997) Extracellular factor(s) following exposure to alpha-particles can cause sister chromatid exchanges in normal human cells. *Cancer Res* **57**: 2164–2171
Lorimore SA, Kadhim MA, Pocock DA, Papworth D, Stevens DL, Goodhead DT, and Wright EG (1998) Chromosomal instability in the descendants of unirradiated surviving cells after α-particle irradiation. *Proc Natl Acad Sci USA* **95**: 5730–5733
Mooney RE (1999) The effect of L-deprenyl on survival and induction of genomic instability in human keratinocytes exposed to gamma-irradiation or chemotherapy drugs, PhD thesis, University of Dublin
Morgan WF, Day JP, Kaplan MI, McGhee EM and Limoli CL (1996) Genomic instability induced by ionizing radiation. *Radiat Res* **146**: 247–258
Mothersill C and Seymour CB (1986) Effect of lactate on the recovery of CHO-KI cells from gamma radiation damage. *Acta Radiol Oncol* **25**: 71–76
Mothersill C and Seymour CB (1997*a*) Medium from irradiated human epithelial cells but not human fibroblasts reduces the clonogenic survival of unirradiated cells. *Int J Radiat Biol* **71**: 421–427
Mothersill C and Seymour CB (1997*b*) Lethal mutations and genomic instability. *Int J Radiat Biol* **71**: 751–758
Mothersill C and Seymour CB (1998*a*) Cell–cell contact during gamma irradiation is not required to induce a bystander effect in normal human keratinocytes: evidence for release during irradiation of a signal controlling survival into the medium. *Radiat Res* **149**: 256–262
Mothersill C and Seymour CB (1998*b*) Mechanisms and implications of genomic instability and other delayed effects of ionizing radiation exposure. *Mutagenesis* **13**: 421–426
Nicholson DW and Thornberry NA (1997) Caspases: killer proteases. *Trends Biochem Sci* **22**: 299–306
Pirisi L, Yasumoto, Feller S, Doniger J and DiPaolo J (1988) Transformation of human fibroblasts and keratinocytes with human papillomavirus type 16 DNA. *J Virol* **61**: 1061–1066
Puck TT and Marcus PI (1956) Action of X-rays on mammalian cells. *J Exp Med* **103**: 653–666
Seymour CB (1983) Radiobiological effects of lactate and glycolysis inhibitors on cultured mammalian cells. PhD thesis, University of Dublin, Trinity College
Seymour CB and Mothersill C (1981) The radiobiological effects of lactate on cells in culture. *Int J Radiat Biol* **40**: 283–293
Seymour CB and Mothersill C (1987) The effect of glycolysis inhibitors on the recovery of CHO-KI cells from split-dose irradiation. *Acta Radiol Oncol* **26**: 367–371
Seymour CB and Mothersill C (1988) The effect of glycolysis inhibitors on the radiation response of CHOI-KI cells. *Radiat Environ Biophys* **27**: 49–57
Seymour CB and Mothersill C (1997) Delayed expression of lethal mutations and genomic instability in the progeny of human epithelial cells that survived in a bystander-killing environment. *Radiat Oncol Invest* **5**: 106–110
Seymour CB, Mothersill C and Moriarty M (1985) Glucose analogues alter the response of CHO-KI cells to gamma irradiation. *Acta Radiol Oncol* **24**: 351–356
Stamato T, Weinstein R, Peters B, Hu J, Doherty B and Giaccia A (1987) Delayed mutation in Chinese hamster cells. *Somatic Cell Mol Genet* **13**: 57–66
Thornberry NA and Lazebnik Y (1998) Caspases: enemies within. *Science* **281**: 1312–1316
Villa P, Kaufmann SH and Earnshaw WC (1997) Caspases and caspase inhibitors. *Trends Biochemi Sci* **22**, 388–393.
Wu L-J, Randers-Pehrson G, Waldren CA, Geard CR, Yu ZL and Hei, TK (1999) Targeted cytoplasmic irradiation with Alpha particles induces mutations in mammalian cells. Proc Natl Acad Sci USA 96 (in press)
Zamzami N, Marchetti P, Castedo M, Hirsch T, Susin SA, Masse B and Kroemer, G (1996) Inhibitors of permeability transition interfere with the disruption of the mitochondrial transmembrane potential during apoptosis. *Fed Eur Biochem Soc Lett* **384**, 53–57.

*© 2000 Cancer Research Campaign*
